# The effect of light and ventilation on house entry by *Anopheles arabiensis* sampled using light traps in Tanzania: an experimental hut study

**DOI:** 10.1186/s12936-022-04063-3

**Published:** 2022-02-05

**Authors:** Arnold S. Mmbando, John Bradley, Deogratius Kazimbaya, Robert Kasubiri, Jakob Knudsen, Doreen Siria, Lorenz von Seidlein, Fredros O. Okumu, Steve W. Lindsay

**Affiliations:** 1 Environmental Health & Ecological Sciences, Ifakara Health Institute, P.O. Box 53, Ifakara, Tanzania; 2 Department of Biosciences, Durham University, Durham, DH13LE UK; 3 MRC International Statistics and Epidemiology Group, London School of Hygiene & Tropical Medicine, London, UK; 4 Royal Danish Academy - Architecture, Design and Conservation, Copenhagen, Denmark; 5 Mahidol-Oxford Tropical Medicine Research Unit (MORU), Faculty of Tropical Medicine, Mahidol University, Bangkok, Thailand; 6 School of Public Health, University of the Witwatersrand, 1 Smuts Avenue, Braamfontein, 2000 Republic of South Africa; 7 Institute of Biodiversity, Animal Health & Comparative Medicine, University of Glasgow, Glasgow, G12 8QQ UK; 8 School of Life Science and Bioengineering, Nelson Mandela African Institution of Science & Technology, P.O. Box 447, Arusha, Tanzania

**Keywords:** *Anopheles arabiensis*, Housing, Light traps, Malaria, Tanzania, Ventilation

## Abstract

**Background:**

In sub-Saharan Africa, house design and ventilation affects the number of malaria mosquito vectors entering houses. This study hypothesized that indoor light from a CDC-light trap, visible from outside a hut, would increase entry of *Anopheles arabiensis*, an important malaria vector, and examined whether ventilation modifies this effect.

**Methods:**

Four inhabited experimental huts, each situated within a large chamber, were used to assess how light and ventilation affect the number of hut-entering mosquitoes in Tanzania. Each night, 300 female laboratory-reared *An. arabiensis* were released inside each chamber for 72 nights. Nightly mosquito collections were made using light traps placed indoors. Temperature and carbon dioxide concentrations were measured using data loggers. Treatments and sleepers were rotated between huts using a randomized block design.

**Results:**

When indoor light was visible outside the huts, there was an 84% increase in the odds of collecting mosquitoes indoors (Odds ratio, OR = 1.84, 95% confidence intervals, 95% CI 1.74–1.95, p < 0.001) compared with when it was not. Although the odds of collecting mosquitoes in huts with closed eaves (OR = 0.54, 95% CI 0.41–0.72, p < 0.001) was less than those with open eaves, few mosquitoes entered either type of well-ventilated hut. The odds of collecting mosquitoes was 99% less in well-ventilated huts, compared with poorly-ventilated traditional huts (OR = 0.01, 95% CI 0.01–0.03, p < 0.001). In well-ventilated huts, indoor temperatures were 1.3 °C (95% CI 0.9–1.7, p < 0.001) cooler, with lower carbon dioxide (CO_2_) levels (mean difference = 97 ppm, 77.8–116.2, p < 0.001) than in poorly-ventilated huts.

**Conclusion:**

Although light visible from outside a hut increased mosquito house entry, good natural ventilation reduces indoor carbon dioxide concentrations, a major mosquito attractant, thereby reducing mosquito-hut entry.

**Supplementary Information:**

The online version contains supplementary material available at 10.1186/s12936-022-04063-3.

## Background

In sub-Saharan Africa, most malaria transmission occurs indoors at night [1, 2]. The design of a house [3, 4], its height above ground [5] and the degree of crowding in a building [6] affect house entry by malaria mosquitoes. One reason for this is that the relative attractiveness of a building depends on how carbon dioxide produced by dwellers emanates from a house [3]. This gas is a major mosquito attractant [7], with large and concentrated plumes being more attractive than low concentrations diffusing out from numerous parts of the building [3]. Ultimately, it may be possible to design ‘stealth’ houses, where few mosquitoes find and enter a house.

Improved ventilation is critically important for reducing malaria transmission inside houses for multiple reasons: (i) it will dilute the carbon dioxide concentration indoors, reducing the odour plumes emanating from a building [4], making it less likely that a blood-seeking mosquito will locate a person to feed on and transmit malaria, (ii) it will keep the bedroom cooler, cooling the body and reducing carbon dioxide production from those sleeping in the room [4], and, (iii) cooling the house makes it more likely that people will sleep under a bed net [8]. Based on the need to keep a house well-ventilated and cool, scientists have designed several prototype houses to reduce mosquito-house entry [9]. The houses were constructed with walls made of shade cloth, which is permeable to both air and light, with a low heat capacity, resulting in rapid cooling of the house at night. A pilot study of six prototype well ventilated houses in Tanzania showed that there was a 95% reduction in mosquito-house entry in double-storey buildings and a 70% reduction in screened single-storey buildings elevated on stilts compared with unmodified reference houses. Both elevated single- and two-storey buildings were 2.3 °C (95% CI 2.2–2.4) cooler than traditional housing. Thus, using materials to construct walls that increased ventilation and had a low thermal mass resulted in few mosquitoes indoors and cooler indoor temperatures. In addition, elevating a house also reduces mosquito entry, as shown by an experimental hut study in The Gambia, where individual huts were raised or lowered to different heights [5].

**Fig. 1:**
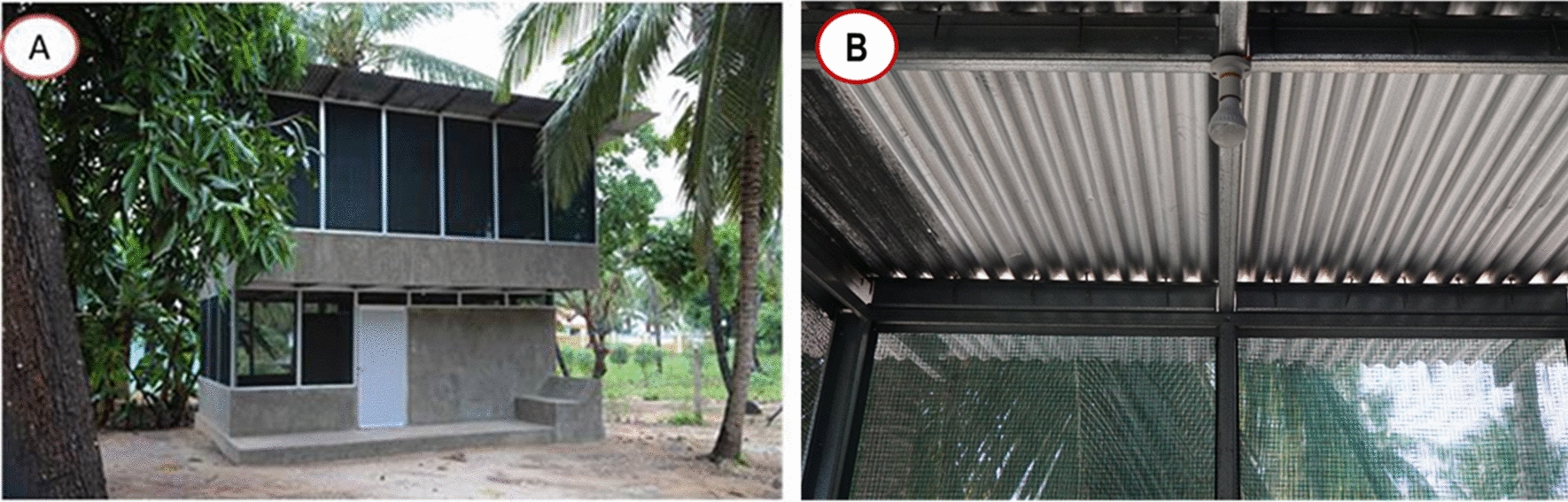
Star home. **A** exterior view; **B** interior view showing the air-permeable green shade-net wall and the bright light above the purlins and below the corrugate iron roof showing the openings under the roof

Based on these encouraging findings, a randomized controlled trial (RCT) exploring the impact on health of a new healthy house, known as a Star home (Fig. 1A), is in progress in rural south-eastern Tanzania [9]. Star homes are two-storey buildings, with the bedrooms on the upper storey and a kitchen and storeroom on the ground storey. The house is designed to be cool and is well ventilated largely because it is constructed using shade-net panels, which are air permeable, for most of the walls. Before starting the trial, however, there were two concerns about the novel house and study design, which lead to the series of experiments reported here. Firstly, it was hypothesised that light from the Centers for Disease Control and Prevention (CDC) light traps used to evaluate the protective efficacy of these houses, would be seen from outside the house and might attract more mosquitoes into the house compared with nights when the trap was not used—inflating mosquito collections in Star homes. Secondly, the Star home has small gaps under the corrugate-roofing sheeting that might be an important entry point for mosquitoes (Fig. 1B), given that open eaves are the major route by which *Anopheles gambiae *sensu lato (*s.l*.) enters traditional houses [10, 11]. The experiments described here, were designed to answer these questions, but simultaneously enabled assessment of how light emanating from the light-traps in combination with improved ventilation affected the house-entering behaviour of *Anopheles arabiensis,* the most common vector of malaria in the Rift valley and drier parts of sub-Saharan Africa [12].

## Methods

### Study area

**Fig. 2:**
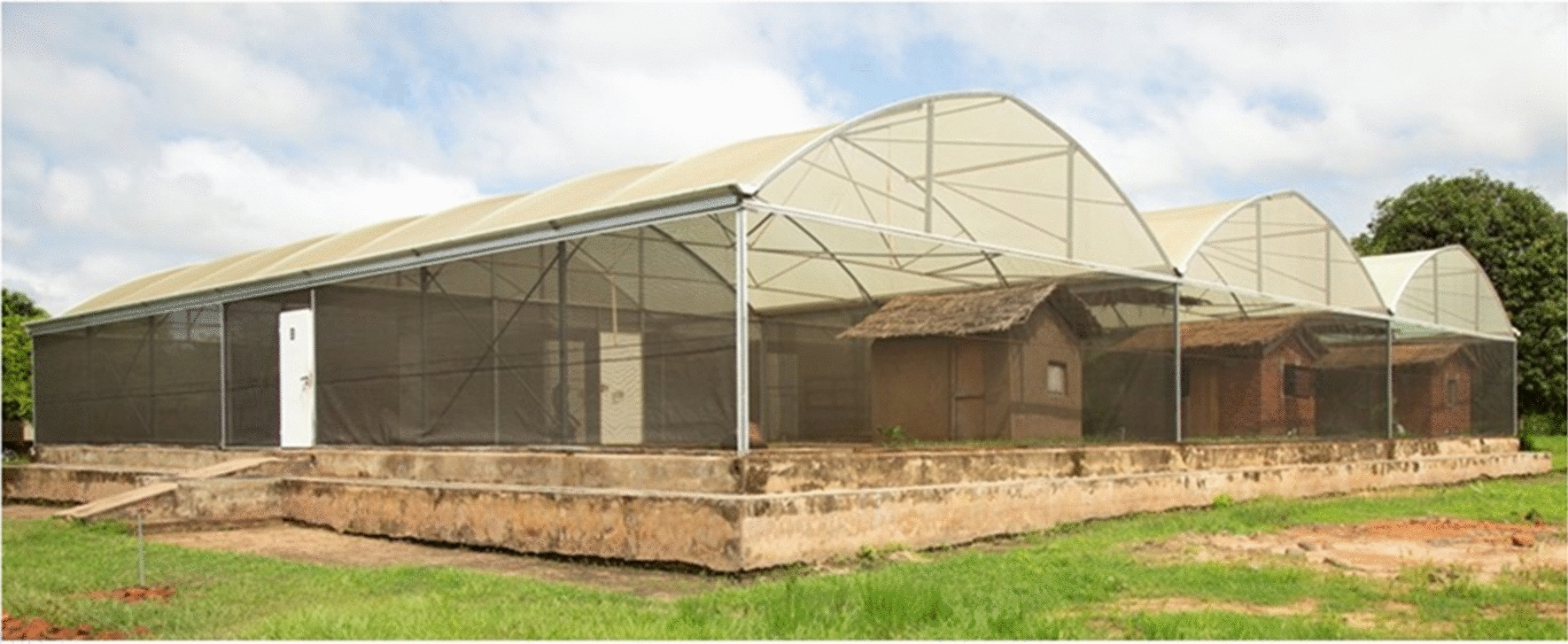
Ifakara Health Institute Semi-field compartments located at the Mosquito City facility in Kining’ina village, with experimental huts in separate cages

The study was conducted at Mosquito City, Ifakara Health Institute’s semi-field system, located near Kining’ina village (8.11417 S, 36.67484 E), approximately 5 km north of Ifakara town, Tanzania, in the dry season, on 72 nights (i.e. 3 experiments × 24 nights) between September 2020 to February 2021 [13, 14] (Fig. 2). Briefly, the semi-field system is a large outdoor cage constructed with a metal-framed shell and mesh walls, supported on a concrete floor 4.53 m high and 553 m^2^ in area (Fig. 2) [15]. The building contains six identical chambers, each 9.6 m × 9.6 m in floor area, with side-walls 4.1 m high, each housing an experimental hut. In each chamber, the floor is covered with local soil to a depth of 400 mm, which allows vegetation to grow inside [14, 16]. Each night, four chambers were used: two with one typology of hut and two with a comparator hut.

### Study design

A randomized block design was used to allocate treatments to the four chambers in four-night blocks. This was a balanced design such that every possible combination of hut typologies had been tested after six blocks, with each hut typology being tested in each chamber for three blocks (Additional file 1: Table S1). At the start of each experiment, one sleeper was randomly allocated to a hut, and then rotated between huts for the following three nights, such that at the end of a four-night block, each man had slept in each hut six times. This design allowed quantification of the effect of the hut typology, adjusting for variation from night to night, sleeper and chamber. For each experiment, light traps were used to collect mosquitoes indoors for four nights each week for six weeks (n = 24 nights).

**Fig. 3:**
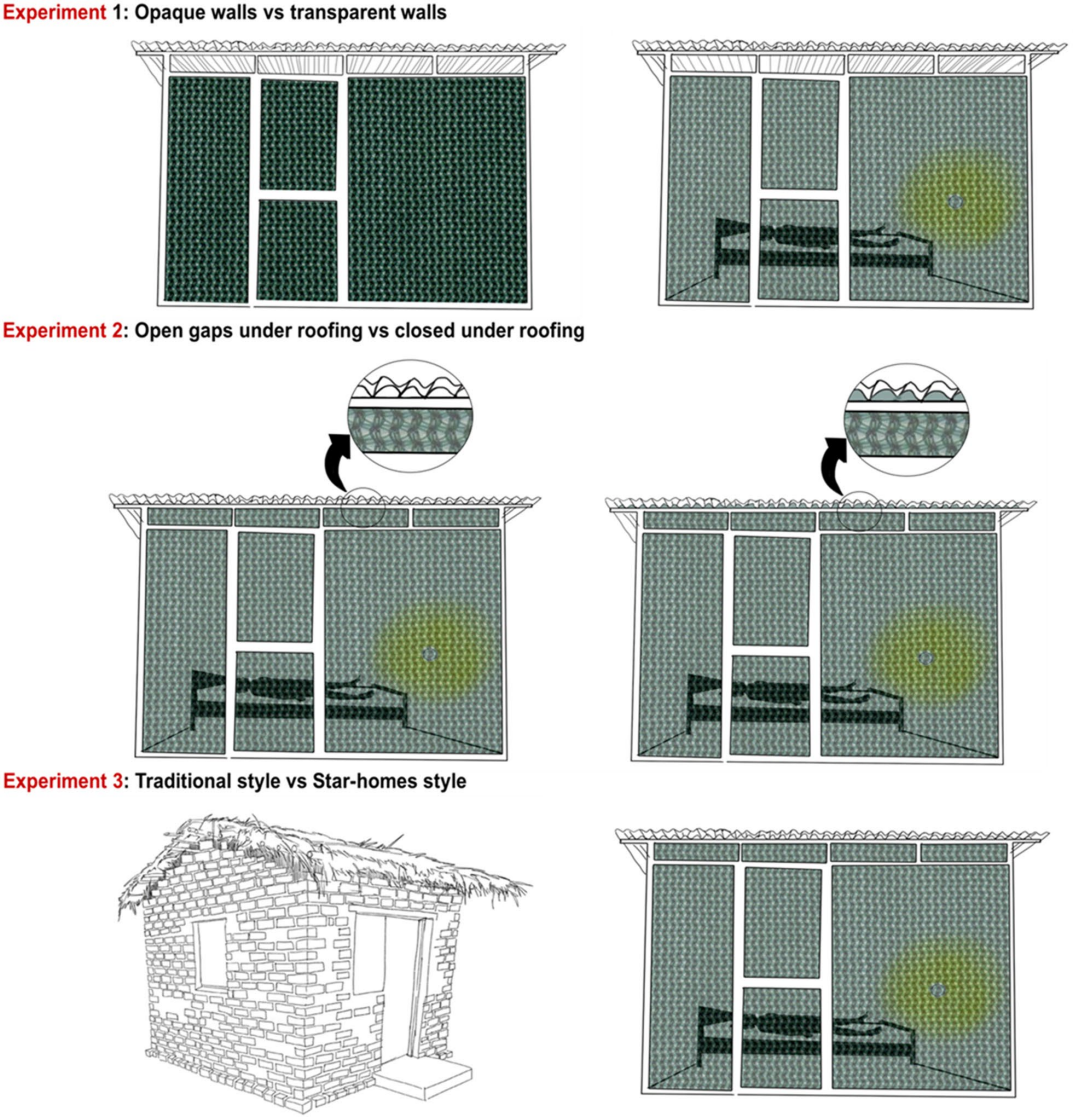
Summary of experiments. The reference hut in each experiment is shown in the first column of each row. In each experiment, local badly-fitting doors were mimicked by adding narrow gaps above and below each door

Three experiments were carried out using four experimental huts each occupied by an adult man. There were two huts in each study group and each hut was situated within a large-screened cage (Fig. 2). Each night 300 *An. arabiensis* female mosquitoes were released in each cage outside the hut and collected indoors using CDC light traps. Experiment 1 compared huts with light-permeable walls with light-opaque walled huts and was designed to assess whether more mosquitoes entered huts with light-permeable walls compared to those with opaque walls. Experiment 2 compared shade-cloth walled huts with openings under the corrugate roof, which mimicked Star homes, with similar huts without holes under the roof. This was designed to determine whether the small-roof openings increased mosquito entry. Experiment 3 compared ‘Star home’ style huts with traditional mud-walled and thatched roof houses, replicating the typologies of housing found in the RCT study area. The experiments are summarized in Fig. 3.

### Experimental huts

Details of the experimental huts are shown in Fig. 3 and in the Additional file 1. In experiment 1, huts with light-permeable walls were compared with light-opaque walled huts. Importantly, the walls consisted of two layers, the external layer was shade cloth and the internal layer clear or opaque plastic. This design compared the effect of light alone, keeping the indoor temperature similar in both hut typologies. Each hut was constructed using 25.4 mm^2^ iron-metal frames and measured 2.62 m × 1.86 m in floor area, with 2.0 m high walls with 150 mm high eave gaps immediately under the over-hanging roof. In each hut, there was one metal door, 1.75 m high and 75 cm wide, with a 20 mm high by 750 mm wide slit above and below the door, to simulate a badly-fitting door common in villages. The roof was made of corrugated sheeting with a sloping flat design.

In experiment 1, light-permeable walls were constructed from panels consisting of 80% shade green nets (high density polyethylene net, Ultra Violet stabilized, Multiknit Ltd, South Africa), with net holes measuring 2 mm × 2 mm on the external face and an internal face of either black opaque high density polythene measuring 2.4 m × 0.69 m × 0.8 mm thick (JK Plastopack Pvt Ltd, Ahmedabad, India) or similar, but clear, transparent plastic sheeting of the same dimensions (Bronze, JK Plastopack Pvt Ltd Ahmedabad, India; Fig. 3). Internal sheeting panels were fixed in place using Velcro strips so that the panels could be moved easily between huts.

In experiment 2, the Star home-style huts were a similar design to those described in experiment 1, although in this experiment there was no internal panels of plastic sheeting, and the eaves gaps were either open or closed. This experiment compared huts with small gaps (24 mm wide and 18 mm radius) which form under the corrugate-roofing panels when lying on purlins (horizontal beams along the length of the roof supporting the rafters), with huts with these roofing holes plugged with sponges (Fig. 3).

In experiment 3, the Star home-style hut described in experiment 2, was compared with a traditional-style hut common in Tanzania and other parts of sub-Saharan Africa, (Fig. 3). The traditional hut had a floor area of 3.1 m × 2.7 m, with walls 1.8 m high. These huts were constructed with burnt-brick walls, 90 mm thick, with gabled, thatched roofs, 50 mm thick, with 200 mm open eaves on all sides of the hut. The hut had one door at the front, 1.75 m high and 0.75 m wide. There were four windows 0.56 m × 0.56 m in size, one on each side of the hut. Although windows were closed during the experiments, they had 5 mm wide gaps on the vertical side of each window to simulate poorly fitting windows.

### Study procedures

Sleepers were recruited to the study after providing their signed consent and tested for malaria parasites using a rapid diagnostic test (paraHIT®f, Span Diagnostics Ltd, Sachin (Surat), India). All tested negative. The men did not smoke, drink alcohol or use perfume during the study.

Three hundred, unfed five-to-eight-day old female laboratory-reared *An. arabiensis* were released in each chamber each night, 3 m from the front door of each hut at 18:30 h. One man, between 18 and 35 years old, slept in each hut under an intact insecticide-treated net (Olyset nets, Sumitomo Chemical, Arusha, Tanzania), measuring 0.9 m wide × 1.8 m long × 1.8 m high. Sleepers entered the huts at 19:00 h and left at 07:00 h the next day. Mosquitoes were collected from each hut using a CDC light trap (incandescent light, Model 512, BioQuip product, California, USA), with the bulb 1 m above the floor at the foot end of the bed and operated from 19.00 h to 07.00 h. Mosquitoes from the light trap were collected and killed by exposure to chloroform. Any remaining mosquitoes were cleared from inside and outside the huts each morning using a mechanical aspirator (Prokopack®, model 1419, John W. Hock Co., Gainesville, USA). Mosquitoes from the light trap and aspirator collections were counted (Additional file 1).

Resting mosquitoes were also collected after the light traps switched off at 07:00 h. All mosquitoes remaining inside the huts and outside were cleared using a mechanical aspirator (Prokopack®, model 1419, John W. Hock Co., Gainesville, USA), each morning from 07:15 h to 07:45 h. Collection of resting mosquitoes started inside the huts, followed by outdoor collections. Resting mosquitoes were collected to ensure no mosquito remained inside the huts and chamber that could affect the next day experiment. Resting mosquitoes collected both inside and outside by using mechanical aspirator were counted and recorded.

Indoor temperature, carbon dioxide concentration and relative humidity were recorded using data loggers (CO_2_Meter.com, model CM-0018-AA, GasLab, Florida, USA). Data loggers were positioned in the centre of each hut, 1 m above the ground, and recorded at 30 min intervals from 18:30 h to 07:00 h. Outdoor temperature, carbon dioxide concentration and relative humidity were measured only in experiment 3, at a height of 1 m high in the centre of each large cage 5 m from each hut.

### Outcomes

The primary outcome was the proportion of host-seeking and resting *An. arabiensis* collected inside each hut each night using light traps and Prokopack aspirators, respectively. Secondary outcomes were mean indoor temperature and mean indoor carbon dioxide concentration recorded between 19.00 h and 07.00 h.

### Data analyses

Sample size was estimated based on a previous study [17] done at the study site where the mean number of *An. arabiensis* collected per trap per night was 10.4 (SD = 21.5). The sample size simulation was based on a negative binomial distribution to detect a 50% reduction in indoor mosquitoes at the 5% level of significance with 90% power, 24 nights (six weeks) of experimentation was sufficient.

Data analysis was done with R (version 3.3.2) [18], using *lme4* [19, 20], and *dplyr* [21] packages*.* Mosquito count data were modelled using generalized linear mixed effect model (*glmer*) using a binomial distribution to account for a log-link function. The recaptured mosquito count numbers per SFS-chamber in a particular night were represented as a proportion of the released mosquitoes in the specific chamber. The response variable was the proportion of mosquitoes caught in light traps, while hut typology was included as fixed factors. The sleeper, chamber ID and nights were included as fixed effects. Model coefficients were exponentiated to obtain the odds ratios (OR) and 95% confidence intervals. Adjusted mean nightly difference of temperature, relative humidity and carbon dioxide concentrations together with its (95% CI) values per night/hut typology were calculated using linear mixed effect model (*lmer*) modelled using a normal distribution. Analysis of variance was used to assess the significance level (p-value) of mean difference of environmental conditions obtained from the huts typology/night. In experiment 3, matched-paired t tests were used to compare the mean indoor carbon dioxide concentrations in each hut type with the mean outdoor concentrations.

## Results

### Experiment 1: Light-opaque walls versus light-transparent walls

During the experiment, 69.5% (10,010/14,400) of mosquitoes released were collected in huts with transparent walls compared with 53.8% (7747/14,400) in those with opaque walls. The average percentage of mosquitoes collected in each hut was 69.9% (95% confidence intervals, CIs 67.4–72.3) in those with transparent walls and 55.8% (95% CIs 52.9–58.6) in those with opaque walls. The adjusted analysis showed that the odds of finding mosquitoes in huts with transparent walls, where the light could be seen from outside, was 84% greater than huts with opaque walls, where little, if any light, was visible from outside (Odds ratio, OR = 1.84, 95% CIs 1.74–1.95, p < 0.001, Table 1). There was no difference in mean nightly indoor temperature or indoor carbon dioxide levels between the two types of hut (Table 3), suggesting that differences in mosquito entry were due to light alone, rather than temperature or carbon dioxide.

**Table 1:** Comparison of indoor densities of malaria vectors between different hut typologies.

Category	Description	Mean no. mosquitoes (%)/night (95%CI)	Odds ratio (95% CI)	*p*-value
Experiment 1: Light-opaque walls vs light-transparent walls				
Typology	Opaque-walled	55.8 (52.9–58.6)	1	< 0.001
	Transparent-walled	69.9 (67.4–72.3)	1.84 (1.74–1.95)	
Experiment 2: Open gaps under roofing vs closed gaps under roofing				
Typology	Open gaps	0.03 (0.01–0.12)	1	< 0.001
	Closed gaps	0.02 (0.00–0.10)	0.54 (0.41–0.72)	
Experiment 3: Poorly ventilated vs well-ventilated				
Typology	Poorly ventilated	19.3 (17–21.9)	1	< 0.001
	Well ventilated	0.3 (0.16–0.66)	0.01 (0.01–0.03)	

Covariates in the model include sleeper, hut position and night

Where *CI* confidence intervals, *OR*  odds ratio

There was no difference in indoor resting *An. arabiensis* collected in the different typologies of houses (Odds ratio = 0.89, 95% CIs 0.74–1.05, p = 0.17). There were fewer outdoor resting *An. arabiensis* in transparent-walled houses compared to opaque-walled houses (OR = 0.57, 95% CIs 0.54–0.64, p < 0.001; Table 2).

**Table 2:** Comparisons of indoor and outdoor resting of malaria vectors between two house types

Experiments		Description	Indoor resting mosquitoes caught by Prokopack® aspirators			Outdoor resting mosquitoes caught by Prokopack® aspirators		
			Mean no. mosquitoes/night (95% CI)	Odds ratio (95%CI)	*p*-value	Mean no. mosquitoes/night (95% CI)	Odds ratio (95%CI	*p-*value
Experiment 1: Light-opaque walls vs light-transparent walls								
	Opaque-walled		1.3 (0.8–2.1)	1		8.1 (6.9–9.5)	1	
	Transparent-walled		1.1 (0.7–1.9)	0.89 (0.74–1.05)	= 0.17	4.9 (4.1–5.8)	0.57 (0.54–0.64)	< 0.001
Experiment 2: Open gaps under roofing vs closed gaps under roofing								
	Open eave-gaps		9.4*e−4 (0.0–0.1)	1		59.1 (56–62)	1	
	Closed eave-gaps		1.7*e−4 (0.0–0.01)	0.19 (0.08–0.44)	< 0.001	60.6 (57.6–63.5)	1.07 (1.02–1.12)	= 0.008
Experiment 3: Poorly ventilated vs well-ventilated								
	Traditional		0.5 (0.3–0.8)	1		50.1 (46.4–53.8)	1	
	Star-homes type		0.1 (0–0.1)	0.12 (0.06–0.23)	< 0.001	75.3 (72.5–78.0)	3.04 (2.90–3.20)	< 0.001

24 nights of experimentations done in each experiment; each house type test was replicated inside two chambers. 300- host-seeking laboratory reared *An. arabiensis* released in each SFS-chamber. Prokopack® aspirators used to collect resting mosquitoes inside and outside the huts

### Experiment 2: Open gaps under roofing versus closed gaps under roofing

During this experiment, just 1.0% (144/14,400) of the mosquitoes released were collected in huts with open gaps under the roofing compared with 0.6% (80/14,400) in those where the gaps were closed. The average percentage of mosquitoes collected in each hut was 0.03% (95% CIs 0.01–0.12) in those with gaps and 0.02% (95% CIs 0.0–0.1) in those without. In the adjusted analysis, 46% fewer mosquitoes were collected in huts with no gaps than those with open gaps (OR = 0.54, 95% CIs 0.41–0.72, p < 0.001) (Table 1). There was no difference in temperature nor carbon dioxide between the two types of hut (Table 3).

**Table 3:** Environmental measurements between the different hut typologies. Where CI = confidence intervals, ppm = parts per million

Variable	Description	Mean (95% CI)	Adjusted mean difference (95% CI)	*p*-value
Experiment 1: Light-opaque walls vs light-transparent walls				
Temperature (°C)	Opaque-walled	27.1 (26.1–28.1)	1	
	Transparent-walled	26.2 (24.8–27.6)	0.9 (0.1–2.4)	= 0.84
Relative humidity (%)	Opaque-walled	59 (56–62)	1	
	Transparent-walled	63 (60–66)	4 (0.4–8)	= 0.27
Carbon dioxide (ppm)	Opaque-walled	414 (394–434)	1	
	Transparent-walled	407 (383–430)	−7 (−21–34)	= 0.80
Experiment 2: Open gaps under roofing vs closed gaps under roofing				
Temperature (°C)	Open gaps	28.3 (28–28.5)	1	
	Closed gaps	28.2 (28–28.5)	−0.1 (−0.8–0.1)	= 0.84
Relative humidity (%)	Open gaps	64.0 (62.8–65.2)	1	
	Closed gaps	65.0 (63.7–66.3)	0.8 (−0–2)	= 0.50
Carbon dioxide (ppm)	Open gaps	398 (387–408)	1	
	Closed gaps	388 (377–399)	−10 (−22–2)	= 0.43
Experiment 3: Poorly ventilated vs well-ventilated				
Temperature (°C)	Traditional	26.1 (25.7–26.4)	1	
	Star-homes types	24.8 (24.6–25.1)	−1.3 (−1.7– −0.9)	< 0.001
Relative humidity (%)	Traditional	74.6 (72.4–76.7)	1	
	Star-homes types	82.2 (81.1–83.3)	7.8 (5.9–9.7)	< 0.001
Carbon dioxide (ppm)	Traditional	541 (516–565)	1	
	Star-homes types	320 (314–327)	−97 (−116–78)	< 0.001

Huts with closed-eaves were less likely to have indoor resting *An. arabiensis* than those with open-eaves (OR = 0.19, 95% CIs 0.08–0.46, p < 0.001). There was a corresponding increase in outdoor-resting mosquitoes in cages with huts with closed eaves compared to cages with open eave huts (OR = 1.03, 95% CIs 0.97–1.09, p < 0.05; Table 2).

### Experiment 3: Poorly ventilated versus well ventilated

In this experiment, only 0.3% (46/14,400) of the mosquitoes released were collected in the well-ventilated Star home-style huts compared with 29.5% (4246/14,400) in the poorly ventilated traditional-style huts. The average percentage of mosquitoes collected in each hut was 0.3% in the Star-home style huts (95% CIs 0.16–0.66) and 19.3% in the traditional-style huts (95% CIs 17.0–21.9). The adjusted analysis showed that the odds of mosquito house entry was 99% less in well-ventilated huts than poorly-ventilated huts (OR = 0.01, 95% CIs 0.01–0.03, p < 0.001, Table 1).

The odds of collecting indoor-resting mosquitoes was 88% less in well-ventilated, Star-home-style huts than traditional-style huts (OR = 0.12, 95% CIs 0.06–0.23, p < 0.001). Consequently, the cages of Star-home style huts had an increased odds of collecting outdoor resting *An. arabiensis* than traditional-style huts (OR = 3.04, 95% CIs 2.90–3.20, p < 0.001; Table 2).

The indoor temperature was 1.3 °C, (95% CIs 0.9–1.7, p < 0.001) cooler in the Star home-style huts (24.8 °C, 95% CIs 24.6–25.1) than traditional-style huts (26.1 °C, 95% CIs 25.7–26.4). There were also lower concentrations of carbon dioxide indoors in Star home-style huts (mean concentration = 320 ppm, 95%, CI 314–327) than traditional-style huts (541 ppm, 95% CI 516.4–565.4, p < 0.001). Importantly, carbon dioxide concentrations in Star home-style huts were similar to outdoor levels (mean difference = 11 ppm, 95% CIs 4–13, p = 0.95), but were 232 ppm higher in the traditional-style huts than outdoors (95% CIs 176–298, p = 0.03; Table 3). The environment conditions between the hut typologies was similar. During this experiment, the mean nightly outdoor temperature (19.00–07.00 h) was 25.1 °C (95% CIs 24.3–27), and carbon dioxide concentration was 309 ppm (95% CIs 290–320).

## Discussion

This series of experiments assessed three aspects of the Star-homes type huts; (1) transparency vs opacity walls, (2) presence vs absence of small eave gaps under the corrugated iron roofs and (3) ventilation achieved through the permeable walls of shade cloth huts. These experiments provide new insights into the effect of light and ventilation on house entry by one of sub-Saharan Africa’s most important malaria vectors, *An. arabiensis*. In this experimental setting, when the light from the CDC-light trap was visible from outside the hut, the odds of catching mosquitoes indoors increased by 84% compared with when the light was not visible from outside. Clearly, in this experiment light and human odours were attracting mosquitoes from outside the inhabited hut. In the 1960s, in the first pioneering studies where light traps were used to collect African mosquitoes, Odetoyinbo showed that light was an essential element of the CDC light trap, since without light the trap collected 95% fewer *An. gambiae s.l.* [22]. Similarly, when Costantini and co-workers used a light trap indoors without a light they collected 63% fewer *An. gambiae s.l.* than a trap with a light bulb [23].

This finding is important for several reasons. Firstly, light traps are a standard sampling tool for collecting indoor mosquitoes during randomized trials of vector control interventions [24, 25]. Whilst this is probably not a concern in most studies where the sampling units are traditional houses with opaque walls and doors, they may bias the sample where screened doors are used, or if used in houses with multiple small openings (e.g. a bamboo house) which allows the light to be viewed from outside a house. In a recent trial in The Gambia, where screened doors were installed in village houses, the number of mosquitoes collected indoors was higher than in the reference group, with solid doors [26]. It seems likely that the Gambian study may have over-estimated the mosquito densities in houses with screened doors because the light from the trap would have been visible from outside the house. Secondly, it also raises concerns about whether light traps should be used in the trial comparing mosquito-house entry in Star homes with traditional houses. Thirdly, these findings beg the question: will domestic lighting increase malaria transmission? The conclusions are mixed, with most studies indicating increased malaria mosquito biting associated with electrification [27–29], perhaps due to people staying outside longer in the night and getting bitten by malaria mosquitoes. In a study in Tanzania, however, houses with electricity had fewer indoor mosquitoes than those without electricity [30]. Since electricity is associated with greater wealth, fewer mosquitoes may be due to better built homes with fewer mosquito entry points than poorer households or the use of mosquito coils [30, 31]. Clearly, further research is needed to clarify whether electric light, including that generated from tungsten and light-emitting diode bulbs, are attractive to mosquitoes and at what light intensity.

Responses of mosquitoes to light are complex, since it will vary according to the time of day, feeding status of the mosquito, as well as the intensity and wavelength of light. At dawn and dusk, under natural conditions, a substantial proportion of indoor-resting *An. gambiae s.l*., including those that are semi-gravid, gravid and bloodfed, are attracted to the faint light from the windows, whilst intense light experienced during the day prevents exiting [32, 33]. Host-seeking mosquitoes are also stimulated to fly by low light intensities at dusk, with this behaviour being under circadian control [34]. Interestingly, feeding can be interrupted for up to four hours when mosquitoes are exposed to bright white light for 10 min at the start of the night [35]. In Brazil, there was a tenfold reduction in *An. gambiae s.l.* (now known to be *An. arabiensis*) inside brightly-lit houses compared to the darkest houses [36]. In Canada, nocturnal blood-questing mosquitoes are attracted to low intensity light, like black, blue and red, rather than high intensity colours like white and yellow [37], suggesting that this behaviour could be related to the choice of darker day-resting sites. In The Gambia, host-seeking mosquitoes also appear to be attracted to large solid objects over distances of 15–20 m [38]. Gillies and Wilkes suggested that the outline of a house or its degree of isolation from other houses or patches of tall vegetation could affect the attractiveness of one house over another. In conclusion, the evidence suggests that while light in the presence of human odours is attractive to host-seeking mosquitoes, the shape and position of a dwelling may also be important.

Small gaps formed where the corrugate-metal roof rested on a purlin and, as seen in experiment 2, resulted in more mosquitoes entering the hut compared with gap-free huts. This is expected since open eaves, the gap between the top of the wall and the roof, are the major route by which *An. gambiae s.l.* enters a house [10, 11, 39]. In this experiment, however, only a few mosquitoes entered the huts, suggesting that the holes might not cause an appreciable rise in mosquitoes in similarly constructed houses, such as the Star homes. The most plausible explanation for this finding is that shade-cloth walled huts attracts fewer mosquitoes as it allows carbon dioxide to rapidly be dissipated from the huts, unlike those with solid-plastic walls used in experiment 1.

In experiment 3, there was a 99% reduction in mosquitoes entering the well ventilated, Star home-type huts, compared to the poorly-ventilated huts, which resembled traditional houses. The principal explanation for this difference in attractiveness is related to the concentration gradient of carbon dioxide leaving the two typologies of hut. In the well-ventilated hut the carbon dioxide concentration was just 11 ppm higher than outdoor levels, illustrating how effectively the gas is removed from the hut through the permeable walls. Since mosquitoes can only detect differences in carbon dioxide concentrations greater than 40 ppm [40], this suggests that they may not be able to readily detect people sleeping in Star-home style huts. In marked contrast, the poorly-ventilated huts have carbon dioxide concentrations considerably higher, 232 ppm above background levels, providing steep concentration gradients of the gas which allows outdoor mosquitoes to locate a host indoors. These findings are supported by a recent study in The Gambia, which showed that a well-ventilated house could reduce indoor mosquito densities by 80% compared with a poorly-ventilated house [4]. The well-ventilated huts also reduced indoor temperature by 1.3 °C compared to the poorly-ventilated hut, which is likely to increase human comfort and, hence, usage of bed nets [9]. Star homes with their well-ventilated walls are likely to act as ‘stealth houses’, especially as the bedrooms are situated on the second storey. Recent research shows that the number of *An. gambiae s.l.* enter an inhabited building declines with increasing height, with 84% fewer mosquitoes when houses are elevated 3 m from the ground [5].

The present study has several limitations. First, the experimental huts were smaller than village houses, so that these findings are unlikely to be directly comparable with the field. Second, only one man slept in each hut, whilst in the village’s two to six people sleep in the same house [41]. Third, the study was conducted in a semi-field system with laboratory-reared *An. arabiensis*, which may differ in their behaviour to wild mosquitoes since colonization is likely to reduce the variation in behavioural traits seen in wild populations. Fourth, the time when the sleepers went to bed was not varied nor were they allowed to open and close the hut door as they chose, behaviours that would influence mosquito-house entry. Fifth, the present study was based on indoor mosquito collections using CDC light traps and it may be that the findings would differ if using sampling techniques that did not use light as an attractant, such as human landing catches.

## Conclusion

Light from a CDC light trap when seen from outside a hut increases the number of host-seeking mosquitoes entering the building compared to a hut with opaque walls. Whilst small gaps under corrugate roofing increase indoor entry, in huts with air-permeable walls, this resulted in few mosquitoes entering the huts. Indeed, the well-ventilated huts had markedly fewer mosquitoes entering the huts compared with traditional dwellings which are hotter and poorly ventilated. Although light traps and holes under the roofing increases the number of mosquitoes entering the building, the presence of air-permeable walls, that increases ventilation, results in remarkably fewer mosquitoes entering the building compared with traditional buildings. The study findings suggest that increasing ventilation in buildings will substantially reduce mosquito entry in Tanzania and is supported by studies from The Gambia [4] suggesting that this may be broadly applicable for malaria control in the region. Considering the absence of other simple sampling tools that are not subject to operator bias, it also suggests that light traps could be used for routine sampling in the Star homes, even though this may slightly over-estimate the true mosquito entry rate. In relation to the design for a healthy house, filling in the small holes under the roofing is likely to make little difference to overall mosquito numbers entering this type of house. Most importantly, the study findings add to the literature suggesting that increasing ventilation in houses in sub-Saharan Africa may contribute to a reduction in malaria transmission and makes bedrooms cooler at night.

## Supplementary Information

**Additional file 1:** **Figure S1.** Huts used in the study; opaque-walled, (A), transparent-walled houses, (B), partial open eave-gaps, (C), completely closed eave-gaps, (D), Star home-style house (E), and traditional-style house (F). **Table S1.** Treatment rotations between the semi-field chambers experiment 1 black fill represents opaque-walled and no fill represents transparent walled, experiment 2 black fill represent open gaps and no fill represent closed gap, and experiment 3 black fill represent poorly ventilated huts and no fill represent well ventilated huts (Star-home style). Each experiment was conducted over 24 nights and the entire project over 72 nights. **Table S2.** Volunteer rotations between semi-field chambers.

## Data Availability

The datasets used for the study are available from the corresponding author on reasonable request.

## Abbreviations

CDC: Centers for Disease Control and Prevention
CO_2_: Carbon dioxide
CI: Confidence intervals
OR: Odds ratio
RCT: Randomized controlled trial
