# Supplementary material for: The effect of light and ventilation on house entry by Anopheles arabiensis sampled using light traps in Tanzania: an experimental hut study
Source: Malar J. 2022 Feb 5;21:36. doi: 10.1186/s12936-022-04063-3 (PMC8818140; doi:10.1186/s12936-022-04063-3)
Supplement: Supplementary file 1 — Additional file 1: Figure S1. Huts used in the study; opaque-walled, (A), transparent-walled houses, (B), partial open eave-gaps, (C), completely closed eave-gaps, (D), Star home-style house (E), and traditional-style house (F). Table S1. Treatment rotations between the semi-field chambers experiment 1 black fill represents opaque-walled and no fill represents transparent walled, experiment 2 black fill represent open gaps and no fill represent closed gap, and experiment 3 black fill represent poorly ventilated huts and no fill represent well ventilated huts (Star-home style). Each experiment was conducted over 24 nights and the entire project over 72 nights. Table S2. Volunteer rotations between semi-field chambers. [file 12936_2022_4063_MOESM1_ESM.docx]

**Supplementary materials**

**The effect of light and ventilation on house entry by *Anopheles arabiensis* sampled using light traps in Tanzania: an experimental hut study**

Arnold S. Mmbando, John Bradley, Deogratius Kazimbaya, Robert Kasubiri, Jakob Knudsen, Doreen Siria, Lorenz von Seidlein, Fredros O. Okumu and Steve W. Lindsay





**Supplementary figure 1:** Huts used in the study; opaque-walled, (A), transparent-walled houses, (B), partial open eave-gaps, (C), completely closed eave-gaps, (D), Star home-style house (E), and traditional-style house (F).

**Supplementary table S1**: Treatment rotations between the semi-field chambers experiment 1 black fill represents opaque-walled and no fill represents transparent walled, experiment 2 black fill represent open gaps and no fill represent closed gap, and experiment 3 black fill represent poorly ventilated huts and no fill represent well ventilated huts (Star-home style). Each experiment was conducted over 24 nights and the entire project over 72 nights.

| **Nights** | **Round** | **Chamber 1** | **Chamber 2** | **Chamber 3** | **Chamber 4** |
| --- | --- | --- | --- | --- | --- |
| 1 – 4 | 1 |  |  |  |  |
| 5 – 8 | 2 |  |  |  |  |
| 9 - 12 | 3 |  |  |  |  |
| 13 - 16 | 4 |  |  |  |  |
| 17 - 20 | 5 |  |  |  |  |
| 21 - 24 | 6 |  |  |  |  |

**Table S2**: Volunteer rotations between semi-field chambers

| **Day of the week** | **Sleeper** | | | |
| --- | --- | --- | --- | --- |
|  | **Chamber 1** | **Chamber 2** | **Chamber 3** | **Chamber 4** |
| Monday | 1 | 2 | 3 | 4 |
| Tuesday | 2 | 3 | 4 | 1 |
| Wednesday | 3 | 4 | 1 | 2 |
| Thursday | 4 | 1 | 2 | 3 |
